# Supplementary figures and images for: 16p12.1 Deletion Orthologs are Expressed in Motile Neural Crest Cells and are Important for Regulating Craniofacial Development in Xenopus laevis
Source: Front Genet. 2022 Mar 24;13:833083. doi: 10.3389/fgene.2022.833083 (PMC8987115; doi:10.3389/fgene.2022.833083)

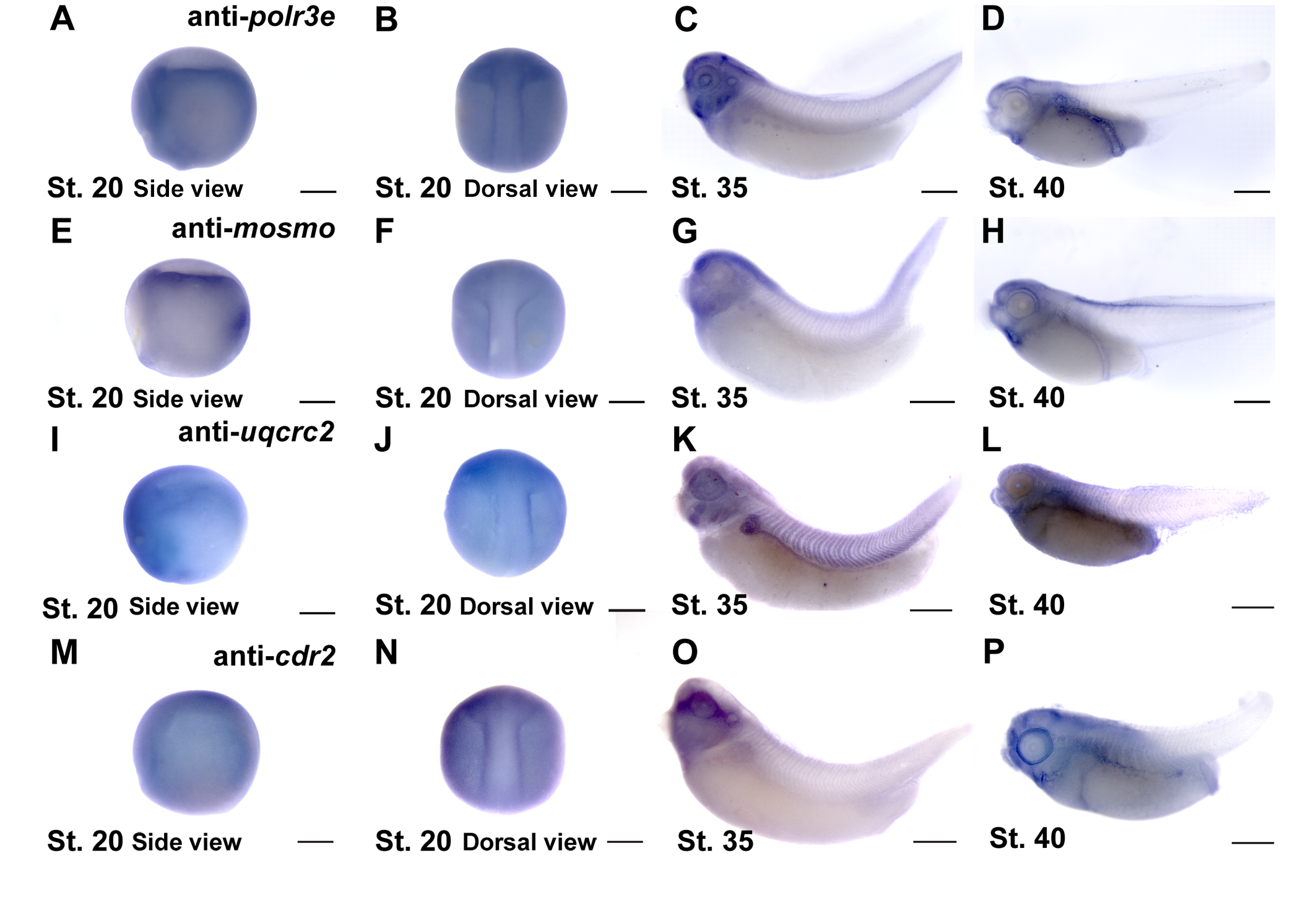

Supplement: Supplementary file 1 [file Image3.TIF]

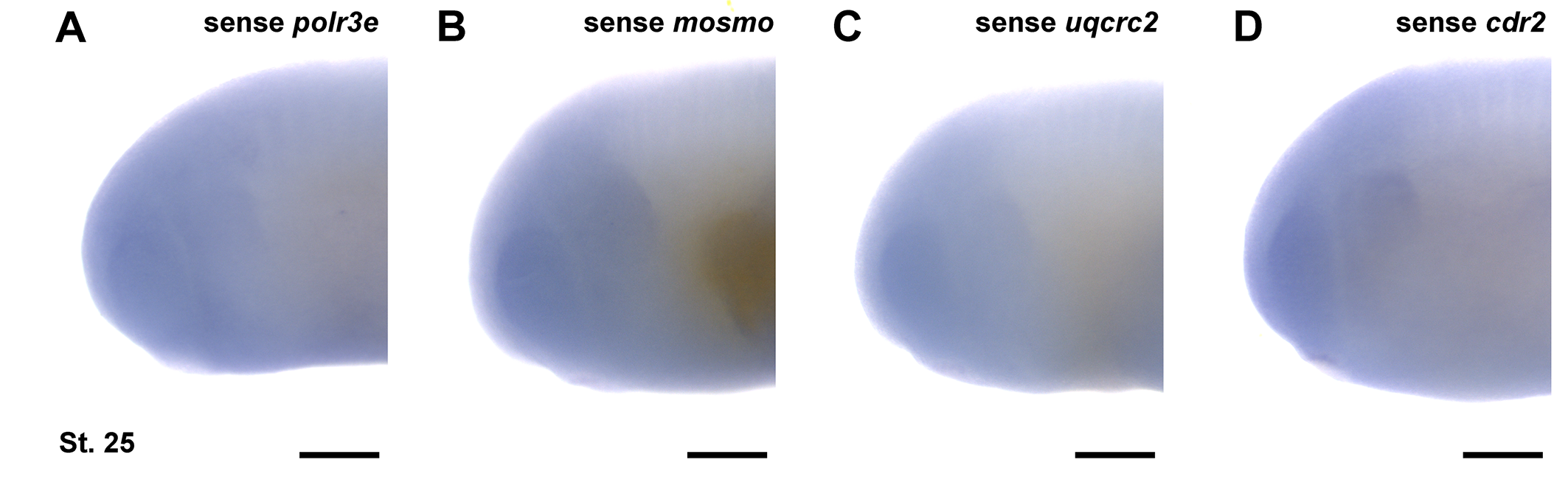

Supplement: Supplementary file 2 [file Image4.TIF]

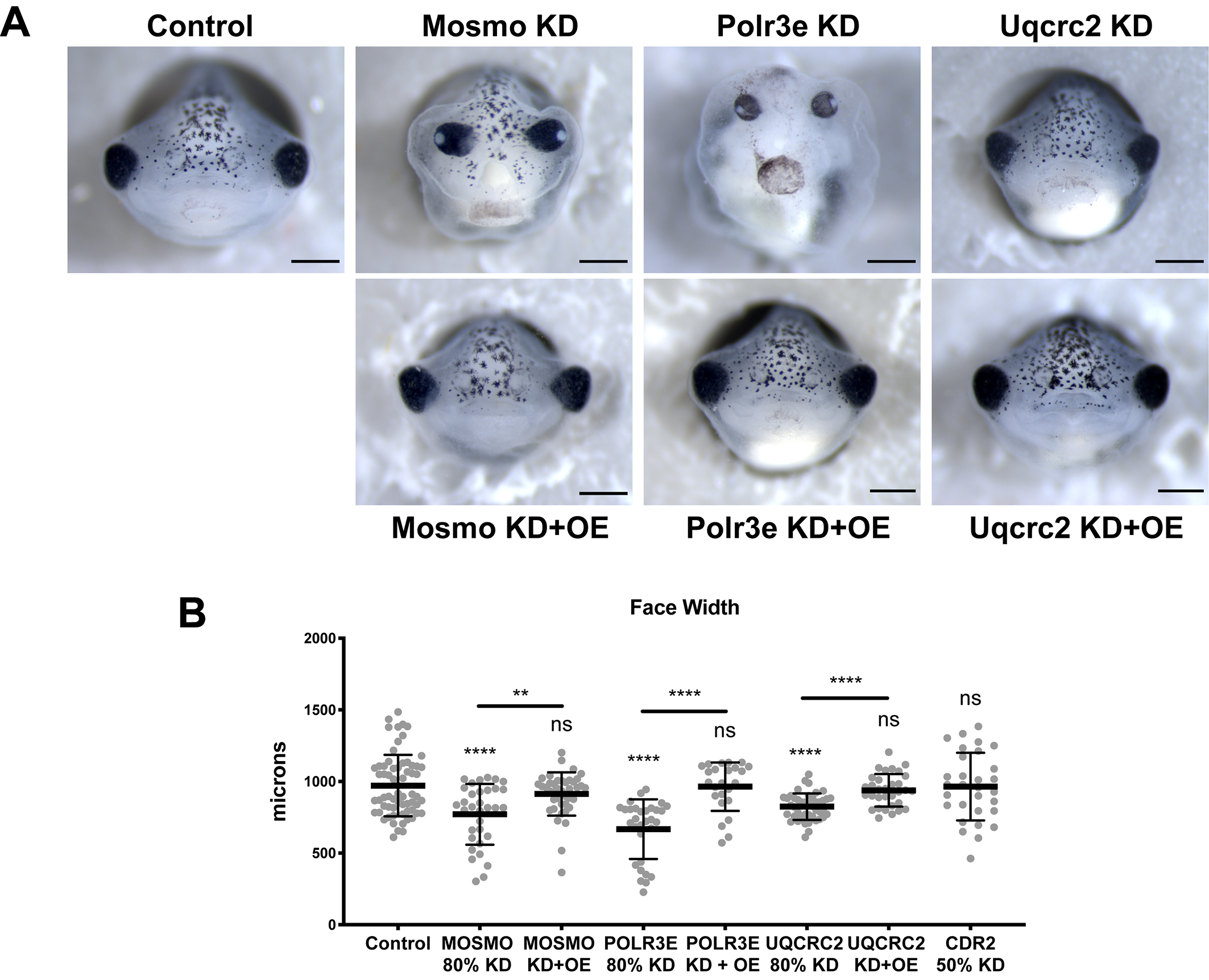

Supplement: Supplementary file 3 [file Image2.TIF]

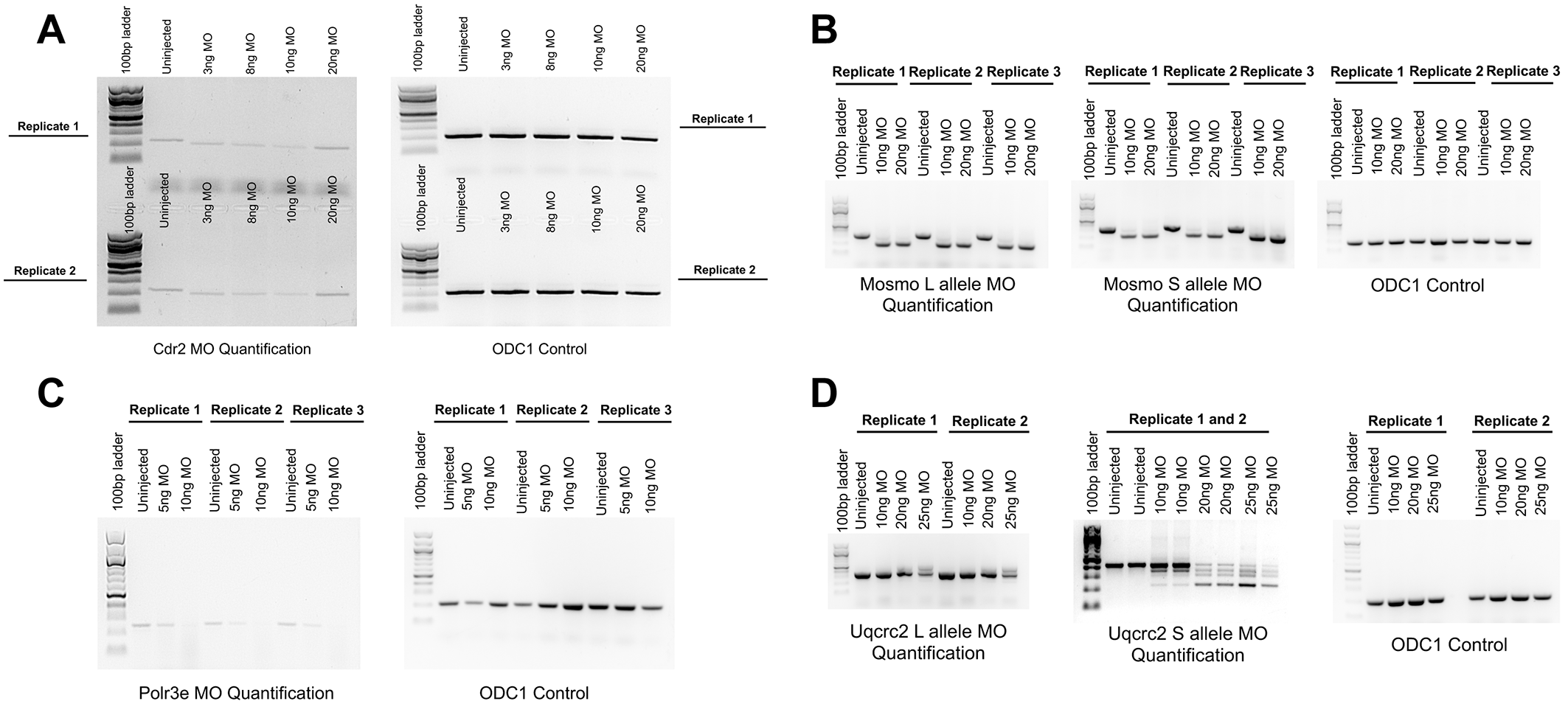

Supplement: Supplementary file 4 [file Image1.TIF]

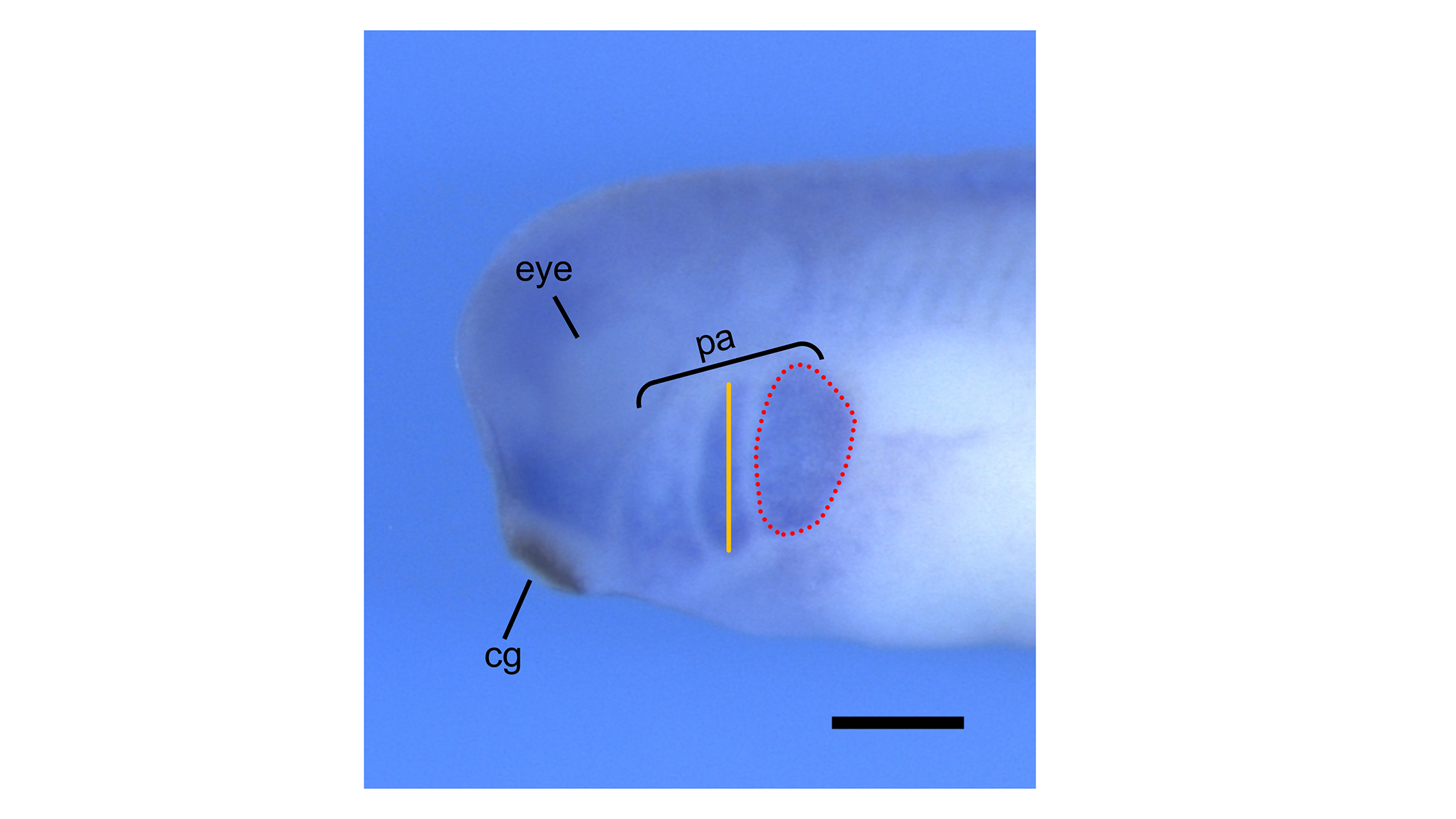

Supplement: Supplementary file 6 [file Image5.TIF]
