# Supplementary material for: 16p12.1 Deletion Orthologs are Expressed in Motile Neural Crest Cells and are Important for Regulating Craniofacial Development in Xenopus laevis
Source: Front Genet. 2022 Mar 24;13:833083. doi: 10.3389/fgene.2022.833083 (PMC8987115; doi:10.3389/fgene.2022.833083)
Supplement: Supplementary file 5 [file DataSheet1.PDF]

Figure S1. Validation of 16p12.1 morpholinos in X. laevis. (A-D) Electrophoretic gels show altered RNA expression of 16p12.1 gene homologs due to splice-site morpholino (MO) knockdown with increasing concentrations in X. laevis embryos. RNA was extracted from 10 two-day old whole embryos for each RT-PCR reaction. At least two replicates (uninjected and multiple MO concentrations) were performed for each MO, and band intensities were compared with expression of ODC1 controls taken from the same cDNA samples and run on gels processed in parallel. Splice site MOs were validated and densitometry was performed to quantify the effectiveness of alternative splicing in our previous manuscript, Pizzo et al., 2021.

Figure S2. Craniofacial defects caused by 16p12.1-associated gene KD are rescued by co-injection of exogenous mRNA co-expression. (A) Row 1: Representative frontal view images of st. 42 tadpoles injected with control MO or MOs for 16p12.1 homologs show defects in craniofacial features, including a decrease in facial width compared to the control. Row 2: Representative frontal view images of st. 42 tadpoles show facial width defects were rescued with co-injection and overexpression of mRNA for each 16p12.1 gene homolog, respectively. **(B)** Boxplot representing the quantification and comparison of facial width measurements with knockdown and overexpression of mRNA of individual 16p12.1 gene homologs normalized to controls. Significance determined using a student's unpaired *t*-test. (Embryos quantified: Control = 65, Polr3e KD = 31, Polr3e KD+OE = 24, Mosmo KD = 33, Mosmo KD+OE = 42, Uqcrc2 KD = 44, Uqcrc2 KD+OE = 32, Cdr2 KD = 30). ****$p < 0.0001$, ***$p < 0.001$, **$p < 0.01$, *$p < 0.05$, n.s., not significant. Scalebar = 500µm.

Figure S3: Expression patterns for 16p12.1-affected genes across early development. *In situ* hybridization utilized **(A-D)** antisense mRNA probe to *polr3e*, **(E-H)** antisense mRNA probe to *mosmo*, **(I-L)** antisense mRNA probe to *uqcrc2*, and **(M-P)** antisense mRNA probe to *cdr2*. Anterior to the left. Lateral and dorsal view images of embryos shown at stage 20 **(A-B, E-F, I-J, M-N)**, lateral view at stage 35 **(C, G, K, O)**, and lateral view at stage 40 **(D, H, L, P)** (n = 10 per probe) Scalebar = 300µm.

Figure S4: *In situ* hybridization probes generated against sense strands of 16p12.1-affected gene mRNAs. In situ hybridization utilized **(A)** sense mRNA probe against *polr3e*, **(B)** sense mRNA probe against *mosmo*, **(C)** sense mRNA probe against *uqcrc2*, and **(D)** sense mRNA probe against *cdr2*, shown at stage 25. (n = 10 per probe). Scalebar = 300µm.

Figure S5: Representative image of stage 28 *X. laevis* embryo labeled for *twist*, a transcription factor that is critical for NCC specification and enriched in the pharyngeal arches. Measurements of the length (orange line) and area (red dotted line) of each individual pharyngeal arch can be done using ImageJ. cg, cement gland; pa, pharyngeal arch. Scale bar = 500µm.
